# Cognitive, neuropsychiatric and neurological alterations in mastocytosis: A systematic review

**DOI:** 10.1002/clt2.12319

**Published:** 2023-12-13

**Authors:** Elena Sagües‐Sesé, Natalia García‐Casares, Ivan Álvarez‐Twose

**Affiliations:** ^1^ Departamento de Medicina, Facultad de Medicina Universidad de Málaga Málaga Spain; ^2^ Centro de Investigaciones Médico‐Sanitarias (CIMES), Fundación General de la Universidad de Málaga, Universidad de Málaga Málaga Spain; ^3^ Instituto de Investigación Biomédica de Málaga y Plataforma en Nanomedicina (IBIMA Plataforma BIONAND) Málaga Spain; ^4^ Instituto de Estudios de Mastocitosis de Castilla‐La Mancha (CLMast) Reference Center for Mastocytosis and CIBERONC Toledo Spain; ^5^ Spanish Network on Mastocytosis (REMA) Toledo and Salamanca Spain; ^6^ Instituto de Investigación Sanitaria de Castilla‐La Mancha (IDISCAM) Toledo Spain

**Keywords:** cognitive impairment, dementia, mastocytosis, neurologic, psychiatric

## Abstract

**Background:**

Mastocytosis manifests with multisystemic symptoms, often involving the nervous system. Numerous cognitive, neuropsychiatric and neurological alterations have been reported in multiple observational studies.

**Methods:**

We performed a qualitative systematic literature review of reported data consulting the electronic databases Medline, Scopus, Web of Science, Cochrane, and BASE until June 2023.

**Results:**

We selected 24 studies in which the majority showed that a high proportion of mastocytosis patients suffer cognitive, neuropsychiatric and neurological alterations. The most common disorders and estimated ranges of frequency observed in adults were depression (68%–75%), anxiety, high stress or irritability (27%–54%), cognitive impairment (27%–39%, primarily affecting memory skills), and headaches (55%–69%). Attention challenges and learning difficulties were reported in children at a rate of 13%, while neurodevelopmental disorders occurred at rates of 8%–12%. Frequent white abnormalities in mastocytosis patients with concomitant psychocognitive symptoms have been reported although neuroimaging studies have been performed rarely in this population.

**Conclusion:**

Further studies with more comprehensive and homogeneous evaluations and neuroimaging and histological analysis should be performed for a better understanding of these manifestations. An earlier detection and proper management of these symptoms could greatly improve the quality of life of these patients.

## 1 INTRODUCTION

Mastocytosis is a clonal disorder characterized by a constitutive activation and abnormal expansion and accumulation of mast cells in one or more organs. This condition is driven by gain‐of‐function somatic mutations in the KIT tyrosine kinase domain.^1^ It is considered a rare disease, with fewer than 50,000 people diagnosed in the United States.^2^ An incidence of 0.89 in 100,000 inhabitants has been reported in a nationwide study of adults in Denmark.^3^ Underdiagnosis is a common concern because of the limited awareness of this condition.^4^ However, increased knowledge of the disease and the availability of more widely accessible diagnostic test in recent years have revealed a higher prevalence of systemic mastocytosis (SM) in adults, estimated at 21 per 100,000 adults in the Verona province.^5^ Children typically not included in these epidemiological reports, as their condition frequently resolves by adulthood.^6^ The diagnosis of mastocytosis is based on well‐established criteria that have been recently revised and updated by the World Health Organization.^7^ According to these criteria, two major forms of mastocytosis are defined: cutaneous mastocytosis (CM) and SM.^8^

Mastocytosis can manifest with a broad spectrum of multisystemic symptoms, depending on the organs infiltrated by neoplastic mast cells and the pattern of proinflammatory mediators released. The reported frequency of affected organs and systems affected in a study of 139 mastocytosis adult patients was as follows: the skin (71% with symptoms such as pruritus, urticarial eruption, angioedema and flushing), the gastrointestinal tract (48% with symptoms like nausea, cramping and diarrhea), the cardiovascular system (36% with symptoms including palpitations, severe anaphylaxis), the musculoskeletal system (27% manifesting primarily with pain), the urinary system (15% with symptoms like pollakiuria and nocturia), and the respiratory system (10% with symptoms including shortness of breath, chest tightness and cough). In contrast, patients frequently experienced neurological, cognitive and psychiatric complaints, with symptoms including headaches (55%), vertigo (32%), irritability (54%), memory loss (52%) to difficulty concentrating in (40%).^9^ Overall, all these symptoms often have a remarkable negative impact on the patients' quality of life.^10^

Mast cell disease exhibits distinct behaviors in pediatric and adult populations. Skin symptoms such as pruritus and flushing are similar in both age groups. Pediatric mastocytosis primarily presents as a cutaneous form, with most cases experiencing spontaneous regression around puberty. In contrast, adult‐onset mastocytosis typically manifests as a systemic disease with a chronic course. In a review of pediatric mastocytosis, the reported frequency of affected systems was as follows: the skin (91% exhibited the Darier sign and 74.8% Urticaria Pigmentosa), the gastrointestinal system (20%), musculoskeletal system (25%) and anaphylaxis (6%).^11^ Neurological abnormalities have not been extensively studied in this population. While some studies suggest an increased frequency of conditions such as autism, attention deficit hyperactivity disorder, and learning difficulties, approximating a frequency of 10%,^12^, ^13^ little is known about other neurological and psychocognitive manifestations in mastocytosis. Moreover, scarce data are available regarding the underlying mechanisms of these alterations. Emerging evidence suggests that brain mast cells may play a role in mood changes, cognition, behavior and stress responsiveness.^14^ Therefore, neurological and psychocognitive impairments may result from the abnormal proliferation of mast cells and their deposition in the nervous system or from the effect of the inflammatory mediators they release.^9^, ^15^

Various established and emerging therapies for mastocytosis, such as targeted drugs against KIT, flavonoids and oral cromolyn, have demonstrated effectiveness in reducing neurological and cognitive symptoms associated with the condition. For instance, the tyrosine‐kinase inhibitor masitinib has not only displayed a high efficiency in vivo by reducing MC burden and tryptase levels,^16^ but has also been shown to decrease depression by 43% compared to baseline.^15^ Additionally, a liposomal luteolin formulation in olive fruit extract has improved attention in children with autism spectrum disorder and alleviated “brain fog” in mastocytosis patients.^17^ Oral cromolyn has proven effective in improving headaches and cognitive symptoms in mastocytosis patients.^18^ Further research and clinical trials involving mastocytosis patients experiencing neurological and psychocognitive alterations may elucidate new therapeutic options.

## 2 MATERIAL AND METHODS

This systematic review was conducted and authored according to the Preferred Reporting Items for Systematic Reviews and Meta‐Analyses (PRISMA) reporting guidelines updated in 2020.^19^

### 2.1 Eligibility criteria

The inclusion criteria of this review were articles or letters to the editor published in peer‐reviewed journals that reported data on neurologic alterations in a human population and/or human specimens, with at least one of the groups having a mastocytosis diagnosis. We excluded case reports, systematic or literature reviews, and conference abstracts.

### 2.2 Information sources

We consulted the electronic databases PubMed/Medline, Scopus/Embase, Web of Science, and the BASE academic search engine until June 2023. Additional sources were bibliographic references of the included studies.

### 2.3 Search strategy

Two independent investigators performed a systematic review of the literature with the keywords “Mastocytosis, UP (cutaneous maculopapular mastocytosis), Cognitive impairment CI, neurologic, cerebral, dementia, psychiatric, psychologic, depression, anxiety, quality, stroke, headache, migraine, epilepsy, seizure, meningitis, encephalopathy, Multiple Sclerosis (MS), Parkinson, Alzheimer, neuropathy, palsy, amyotrophic lateral sclerosis, myopathy, syncope, autism, attention deficit hyperactivity disorder” combined in the following search code: (Mastocytosis OR UP) AND (cogniti* OR neuro* OR cerebral OR dementia OR psychiatr* OR psycho* OR etc.)

### 2.4 Selection process

Two independent reviewers, without the use of any automated tools, assessed the studies to determine their eligibility. We initially selected articles based on a review of their titles and abstracts, aligning them with the objectives and eligibility criteria of this review. Subsequently, a thorough full‐text evaluation was conducted before including them in the review.

### 2.5 Data collection and data items

Data are manually collected in a word document table by two independent reviewers without the use of any automated tools. We extracted the following items: the first author, year of publication, study design, sample size, average age, groups and the evaluations conducted, including cognitive, psychologic and neurological tests, neuroimaging, and serum or bone marrow (BM) mastocytosis biomarkers. Outcome variables included the frequency of disorders reported on the population or the average scores on the tests administered.

## 3 RESULTS

### 3.1 Studies selection

**FIGURE 1:**
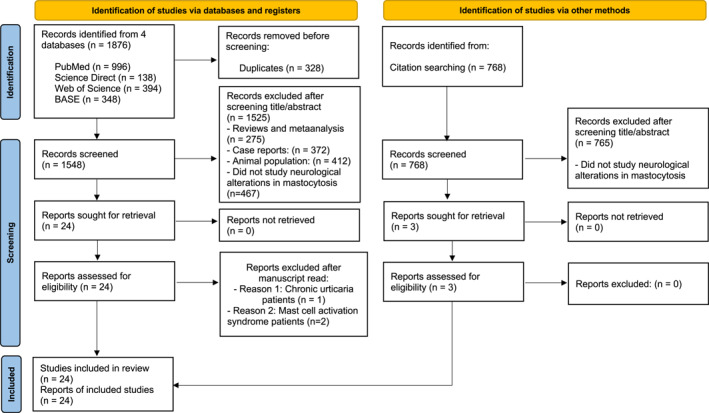
Flow diagram of the study's selection.

The initial search yielded a total of 1876 records: 996 from PubMed/Medline, 138 from Scopus/Embase, 394 from Web of Science and 348 from the BASE. No records were retrieved from Cochrane. A total of 328 duplicate records were removed. Following a review of the titles and abstracts, 1526 results were excluded for the following reasons: 275 were reviews or metaanalysis, 372 were case reports, 412 involved animal study populations, and the remaining did not align with the study's objective, as they did not report neurological alterations in mastocytosis. We initially selected 24 articles^9^, ^10^, ^12^, ^14^, ^15^, ^20^, ^21^, ^22^, ^23^, ^24^, ^25^, ^26^, ^27^, ^28^, ^29^, ^30^, ^31^, ^32^, ^33^ for eligibility assessment. After a full text review, three articles were excluded because the patients in those studies did not have a diagnosis of mastocytosis. Specifically, one article involved only patients with chronic urticaria,^32^ while two papers focused on patients with MC activation syndrome.^20^, ^21^ Additionally, bibliographic references were screened, and three additional articles^13^, ^34^, ^35^ met the inclusion criteria. Consequently, a total of 24 studies^9^, ^10^, ^12^, ^13^, ^14^, ^15^, ^22^, ^23^, ^24^, ^25^, ^26^, ^27^, ^28^, ^29^, ^30^, ^31^, ^33^, ^34^, ^35^, ^36^, ^37^, ^38^, ^39^, ^40^, ^41^ were ultimately selected for inclusion in our review. Figure 1 shows the flow diagram illustrating the study selection process.

### 3.2 Studies characteristics

Table 1 provides a summary of the 25 eligible studies, including their key characteristics and results. The sample sizes ranged from 9 to 400 participants, with an average age of 38 years. Most studies focused on adults with mastocytosis, while only five studies included children aged between 4 months and 12 years.^12^, ^13^, ^25^, ^29^, ^37^ Typically, authors conducted their studies either prospectively or retrospectively, focusing solely on patients diagnosed with mastocytosis, and compared their findings with data from the general population to draw their conclusions. However, some studies also included a control group for comparison.^23^, ^30^, ^36^, ^38^ The diagnosis and subtype classification of mastocytosis typically adhere to the World Health Organization criteria. In most cases, participants underwent assessments for serum tryptase levels, proinflammatory molecules, and KIT mutations in the skin and/or BM, aiming for an accurate diagnostic classification and exploring potential relationships between these factors and the extent of neurological and psychocognitive alterations. Cutaneous forms were typically confirmed through skin histological examination. However, children with CM often did not undergo extensive analysis if clinical criteria were sufficient, as supported by other authors.^42^ Less commonly, the authors investigated whether patients showed improvement while on antihistamine treatment.^15^, ^24^ Additionally, in some studies, patients were selected because of the previously reported neuropsychiatric symptomatology^23^, ^26^ or a neuroinflammatory disorder diagnosis.^31^ The objective was to define their characteristics and identify correlations with biochemical or neuroimaging patterns. Only one of the studies assessed leukocyte telomere length and telomerase activity^14^ to explore potential correlations with the patients' stress levels.

**TABLE 1:** Characteristics and results of the studies.

Author and year	Sample size	Mean age (years)	Study design	Mastocytosis diagnostic exams	Cognitive, neuropsychiatric and quality of life tests	Neuro image	Results	Conclusions
Adults								
Jendoubi F et al 2021^9^	*N* = 139	48	CS (cross‐sectional) Groups: SM (systemic mastocytosis) (*N* = 113) CM (cutaneous mastocytosis) (*N* = 26)	Serum and BM (bone marrow) tryptase Skin biopsy KIT mutation skin and/or BM	Quality of life questionnaire HDRS‐17 (Hamilton depression rating scale)	‐	33.3% M (mastocytosis) patients had mild, 7.4% moderate and 5.6% severe depression. 55% reported headaches, 32% vertigo, 54% irritability, 54% memory loss and 40% difficulty concentrating	Psychocognitive and neurological manifestations are frequent in M
Boddaert N et al 2017^23^	*N* = 72	42	CS Groups: C (controls) (*N* = 33) CM (*N* = 12) ISM (isolated SM) (*N* = 26) SSM (smoldering SM) (*N* = 1)	Serum tryptase KIT mutation skin and/or BM	Neuropsychiatric interview SCL‐90‐R (symptom check list 90R) Modified fatigue index scale	MRI: DTI (diffusion tensor imaging)	MD (major depression) was 30.8% of neuropsychiatric complaints. Morphological brain abnormalities, mainly white matter abnormalities, were found in 49% of M patients with neuropsychiatric complaints, associated with increased perfusion in the putamen, which negatively correlated with depression subscores	Depression is common in M and brain morphological abnormalities appear in almost half of the symptomatic cases
Broesby‐Olsen S et al 2016^36^	*N* = 687,687	‐	CS Groups: C (*N* = 68,700) ISM (*N* = 528) SM/UP (urticaria pigmentosa) (*N* = 116) ASM (aggressive SM) (*N* = 13) SM‐AHNMD (SM with associated hematological non‐mast cell lineage disease (*N* = 23) MCL (mast cell leukemia) (*N* = 7)	‐	Stroke occurrence rate	‐	The rate of stroke was 0.49% (95% CI 0.33–0.67) in the M cohort compared to 0.35% (0.34–0.37) in the controls (HR 1.6% and 10‐year AR 4.6%)	M Patients are at increased risk of stroke
Smith JH et al 2011^26^	*N* = 223	54	Retrospective cohort study Groups: M (*N* = 171) UP (*N* = 52)	‐	Review of neurologic consultations by the patients	‐	30 of 223 M patients with neuropsychiatric complaints had neurologic alterations (13.45%): Syncope (*n* = 12), compression fracture (*n* = 9), compression myelopathy (*n* = 1), migraine (*n* = 5) and MS (multiple sclerosis) (*n* = 3)	Neurological alterations are frequent in M
Smith JH et al 2011^28^	*N* = 64	56	Retrospective cohort study Groups: ISM (*N* = 60) ASM (*N* = 1) SM‐AHNMD (*N* = 3)	KIT mutation BM	Detailed headache survey, HDI (headache disability inventory)	‐	56.2% of M patients had headaches: 37.5% migraine and 17.2% tensional. Headaches associated with M flairs occurred more frequently in males (*p* = 0.002), with higher HDI scores (*p* = 0.02), more histaminergic symptoms and unilateral cranial autonomic features Concurrence of aura with headaches was five‐fold higher than expected	Headache, especially migraine, is common in M
Hermans M et al 2016^34^	*N* = 136	49	Retrospective cohort study Groups: ISM (*N* = 124) ASM (*N* = 7) SM‐AHNMD (*N* = 5)	Serum tryptaseKIT mutation BM	Neuropsychiatric diagnosis of the patients	‐	17 of the patients (12.5%) had neuropsychiatric symptoms: Depression (*n* = 7), CI (*n* = 5), anxiety (*n* = 3), schizophrenia (*n* = 1), ADHD (attention deficit and hyperactivity disorder) (*n* = 1)	Psychocognitive manifestations are frequent in M
Escribano L et al 2009^35^	*N* = 145	37	Prospective cohort study Groups: ISM (*N* = 145)	Serum tryptase Skin biopsy KIT mutation in BM	Neuropsychiatric diagnosis of the patients	‐	34 of M patients had neuropsychiatric symptoms (23.45%)	Neuropsychiatric complications are frequent in M
Vermeiren MR et al 2020^10^	*N* = 50	54	CS Groups: M (*N* = 54) C: from other study	Serum tryptase	SCL‐90‐R SF‐36 (short form health survey)	‐	M Patients scored significantly worse than reported controls in depression, somatization, inadequacy of thinking, acting, and sleeping problems items (*p* = 0.04, 0.00002, 0.002, 0.001, respectively)	Neuropsychiatric complications are frequent in M
Moura DS et al 2011^15^	*N* = 288	47	CS Groups: M (*N* = 288)	‐	HDRS‐17	‐	56% of M patients had mild and 8% severe depression. Masitinib therapy improved symptoms in 67% of the cases (*p* = 0.0001)	Depression is a frequent complication of M
Moura DS et al 2012^24^	*N* = 57	45	CS Groups: M (*N* = 57)	‐	HDRS‐17 WMS (wechsler memory scale)	‐	38.6% patients with M had CI, being working memory and auditory immediate memory the most frequent alterations. 68% patients with M had depressive symptoms (HDRS‐17 > 12)	Psychocognitive manifestations are frequent in M
Rogers MP et al 1986^27^	*N* = 10	49	CS Groups: M (*N* = 10)	Urine histamine or skin biopsy	Psychiatric interview WMS Digit span test Continuous performance test Mood scale	‐	6 M patients had diminished attention span, 7 memory impairment and 6 both; 9 irritability, 3 depression and 6 wide fluctuations in mood, being anger and fatigue the most frequent	Cognitive and affective changes are frequent in M
Spolak‐Bobryk N et al 2022^41^	*N* = 79	‐	CS Groups: ISM (*N* = 54), CM (*N* = 2), MPCM (maculopapular CM) (*N* = 20), SM (*N* = 2), BMM (bone marrow M) (*N* = 1)	Serum tryptase KIT mutation BM Skin biopsy (CM)	MMSE WHO performance status	‐	86% of the patients declared subjective CI, being only 7.6% significantly and 27% slightly relevant in the MMSE, correlating with the percentage of atypical MCs and bone pain	CI is a frequent complication of M
Nicoloro‐santaBarbara J et al 2017^22^	*N* = 180	50	CS Groups: ISM (*N* = 74) CM (*N* = 36) MCAS (mast cell activation sdr) (*N* = 35) SM‐AHNMD (*N* = 17) ASM (*N* = 12) Other (*N* = 6)	‐	MCDQ (mast cell disorder questionnaire) Ways of coping questionnaire Center for epidemiological studies depression scale	‐	64% patients with M had depression, differing the prevalence by income (*p* < 0.001), employment (*p* < 0.001) and education (*p* < 0.05)	Depression is a frequent complication of M
Mesa RA et al 2022^39^	*N* = 119	‐	CS Groups: ISM (*N* = 119)	KIT mutation BM	TouchStone HCP survey	‐	Most patients with ISM felt at least quite a bit depressed, anxious or discouraged because of their condition and life's impact	Depression and anxiety are frequent complications of M
Hermine O et al 2008^38^	*N* = 453	‐	CS Groups: C (*N* = 90) CM (*N* = 46) SM (*N* = 126) Non‐classified (*N* = 101)	Serum tryptase KIT mutation BM Skin biopsy (CM)	Multidimensional questionnaire HDRS‐17	‐	75% of M patients had depressive symptoms (HDRS‐17 > 10), 32% reported a low quality of life, 52% decreased performance status, 55% difficulty with social interactions, 66% memory loss, 69% headache and 20% pain	Psychocognitive and neurological manifestations are frequent in M
Georgin‐Lavialle S et al 2014^14^	*N* = 19	44	CS Groups: ISM (*N* = 13) CM (*N* = 2) ASM (*N* = 4)	Leukocyte telomere length telomerase activity Serum tryptase KIT mutation skin and/or BM	BDI‐II Perceived stress scale	‐	36.8% of M patients had mild and 42.11% moderate‐severe depression. 42.11% perceived high stress, being negatively correlated with telomere length and KIT mutation presence (*r* = −0.728; *p* = 0.001 and *r* = −0.610, *p* = 0.009 respectively)	Neuropsychiatric complications are frequent in M
Georgin‐Lavialle S et al 2016^30^	*N* = 108	50	CS C (*N* = 54) CM (*N* = 2) ISM (*N* = 46) ASM (*N* = 4) SM‐AHNMD (*N* = 2)	Serum tryptase and inflammatory markers KIT mutation skin and/or BM	BDI‐II (Beck Depression Inventory) Perceived stress scale	‐	46.3% of M patients had high stress and 29.6% mild depression, being the score negatively correlated with serum tryptase levels (*p* < 0.0001) and positively correlated with plasma IDO1 activity (*p* < 0.0001)	Neuropsychiatric complications are frequent in M
Rodrigues F et al 2019^31^	*N* = 9	52	CS Groups: M + NID (*N* = 9)	CSF analysis KIT mutation	Neurological assessments	MRI	5 patients had neurological symptoms (3 sensorial and 4 motor), 1 bilateral optic neuritis and 2 psychiatric features, being concomitant MRI subcortical alterations frequently present. MS prevalence in M seems higher than in the general population	Neuroinflammatory disorders may be related to M
Spolak‐Bobryk N et al 2022^40^	*N* = 85	46	CS Groups: CM (*N* = 19) SM (*N* = 66)	Serum tryptase KIT mutation BM Skin biopsy (CM)	Hospital depression and anxiety scale MC‐QoL (mast cell quality of life questionnaire) Cantril ladder	‐	Patients with allergy symptoms presented lower QoL. 27.1% of participants experience anxiety, 12.9% depression, 15.3% low satisfaction with the current life, and 10.6% low satisfaction with life in the next 4 weeks.	Neuropsychiatric complications are frequent in M
Children								
McFarlin KE et al 1991^25^	*N* = 12	3	CS Groups: M (*N* = 12)	‐	Behavior checklist	‐	Mean behavior scores for children with M didn't differ significantly from general population	Behavioral disturbances aren't increased in M children
Gurnee EA et al 2020^29^	*N* = 227	6	CS Groups: MPCM (*N* = 227)	‐	History of ADHD, developmental delay, ASD (austism spectrum disorders) or CI	‐	15 patients with M (8.2%) overall were diagnosed with a neurodevelopmental disorder, being the risk higher in patients between ages 3 and 18 years	Neuro‐developmental disorders aren't increased in M children
Theohari‐des TC et al 2009^12^	*N* = 400	12	CS Groups: M (*N* = 400)	‐	ASD previous diagnosis	‐	41 patients with M presented ASD (10.25%), being Asperger's disorder the predominant diagnosis	ASD is common in M children
Seamens a et al 2016^13^	*N* = 67	11	CS Groups: CM (*N* = 67)	‐	Colorado learning difficulties questionnaire	‐	1 child had ADHD and another ASD, having 7 received therapy. Learning difficulties scores were frequently above the cutoff (13%) in both M children with and without CI	CI is common in M children
Barnes M et al 2014^37^	*N* = 67	0.3	CS Groups: MPCM (*N* = 67)	Serum tryptase Darier sign or skin biopsy	SCORMA index	‐	8% of M patients had headaches and 6% irritability. The number of skin lesions correlated with the systemic symptoms	Headaches and irritability are frequent in M children

Abbreviations: ADHD, Attention deficit/hyperactivity disorder; ASD, Autism spectrum disorder; ASM, Aggressive systemic mastocytosis; BDI‐II, Beck Depression Inventory; BM, Bone Marrow; BMM, Bone marrow mastocytosis; C, Control group; CI, Cognitive impairment; CM, Cutaneous Mastocytosis; CS, cross‐sectional; DTI, Diffusion tensor imaging; HDI, Headache disability inventory; HDRS‐17, Hamilton Depression Rating Scale; IDO1, Indoleamine 2,3‐dioxygenase 1; ISM, Indolent systemic mastocytosis; L, Longitudinal; M, Mastocytosis; MC, Mast cell; MD, Major depression; MCAS, Mast cell activation syndrome; MCDQ, Mast Cell Disorder Questionnaire; MCL, mast cell leukemia; MC‐QoL, Mast cell quality of life questionnaire; MMSE, Mini‐Mental State Examination; MPCM, Maculopapular cutaneous mastocytosis; MRI, Magnetic Resonance Imaging; MS, Multiple Sclerosis; *N*, total number; NID, Neuroinflammatory disorder; PB, Peripheral blood; SCL‐90‐R, SymptomCheck List 90R; SCORMA index, Scoring mastocytosis index; SF‐36, Short form health survey 36; SM‐AHNMD, Systemic mastocytosis with associated hematological non‐mast cell lineage disease; SSM, Smoldering Systemic Mastocystosis; ST, Systemic Mastocytosis; UP, Urticaria Pigmentosa; TDI‐score, Total olfactory function score; WMAs, White matter abnormalities; WMS, Wechsler Memory Scale.

In this review, we have identified the tests conducted in each of the studies for neurological, cognitive and neuropsychiatric evaluations, as well as neuroimaging scans. Neuropsychiatric evaluations encompassed a range of assessments, from psychiatric interviews^23^, ^27^, ^31^ to depression questionnaires^9^, ^10^, ^14^, ^15^, ^22^, ^23^, ^24^, ^30^ and quality of life^9^, ^22^, ^23^ scores. The MC Quality of Life Questionnaire^43^ was conducted only in one of the studies.^40^ Cognitive tests focused on memory^24^, ^27^ and CI.^13^, ^27^ In neurological evaluations, only one of the studies utilized the Minimental State Exam,^33^ while other authors reviewed the patients' previous diagnoses or complaints.^12^, ^26^, ^34^, ^35^ Concerning neuroimaging scans, only two studies performed magnetic resonance imaging,^23^, ^31^ one of them doing diffusion tensor imaging.^23^

### 3.3 Results of the studies

Our primary outcomes of interest encompassed cognitive and neuropsychiatric test results, along with the frequency of diagnosis of neurological or psychiatric disorders in the mastocytosis patient cohort, compared with controls or the general population. Overall, the frequency of cognitive and neuropsychiatric complications is considerably higher in patients with mastocytosis. In adults, the most frequently reported disorders included depression^9^, ^10^, ^14^, ^15^, ^22^, ^24^, ^27^, ^30^, ^34^, ^38^, ^39^, ^40^, anxiety, high stress, or irritability^9^, ^27^, ^30^, ^34^, ^39^, ^40^, CI,^9^, ^13^, ^24^, ^27^, ^29^, ^41^ and headaches.^9^, ^28^, ^37^, ^38^ Among children, reports included attention deficit hyperactivity disorder,^29^ autism spectrum disorder,^12^, ^29^ and learning difficulties.^13^

#### 3.3.1 Cognitive and neuropsychiatric manifestations in adult mastocytosis

The frequency of depression in patients with mastocytosis varied depending on the scale and cutoffs used but was generally notably high. The rates of depressive symptoms ranged from 68% to 75%,^15^, ^38^ mild depression from 29% to 64%,^9^, ^14^, ^15^, ^30^ and moderate to severe depression from 5% to 42%.^9^, ^14^, ^15^ The most frequently reported symptoms included somatization and issues related to thinking, acting and sleeping.^10^ Certain variables showed positive correlations with depressive symptoms, such as income, employment and educational level.^22^ Lower serum tryptase levels and higher indoleamine 2,3‐dioxygenase 1 activity were significantly correlated with more severe depression.^30^ In association, Moura et al^15^ reported that masitinib therapy improved depressive symptoms.

The frequency of anxiety, high stress and irritability were similarly high among mastocytosis patients. Anxiety was reported in up to 27% of patients,^40^ stress in 42%–46%^14^, ^30^ and irritability in 54%.^9^ Wide mood fluctuatioikns were also especially prevalent, with anger and fatigue being the most frequent manifestations.^27^ Georgin‐Lavialle S et al^14^ reported that telomere length correlated with perceived stress, and that patients without KIT mutation showed significantly lower stress levels.

The frequency of CI in patients with mastocytosis has been reported in up to 39% in a study including a total of 57 patients,^24^ where memory loss, difficulty concentrating, and attention span were the most frequent alterations.^9^, ^24^ In another study, the percentage of atypical mast cells and the bone pain degree, but not serum tryptase levels, were found to correlate with the Minimental score decrease.^33^

#### 3.3.2 Cognitive and neuropsychiatric manifestations in pediatric mastocytosis

In the context of pediatric mastocytosis, research suggests that the frequency of autism spectrum disorders is significantly higher, potentially up to 10 times more than the general population, as documented by Theoharides et al. in a comprehensive study involving 400 children with mastocytosis^12^; Within this study, Asperger's syndrome emerged as the most prevalent form among the patient cohort. Additionally, another study showed that 2 out of 17 (12%) children with CM exhibited attention deficit hyperactivity disorder and autism, respectively.^13^ Furthermore, irritability was reported in as many as 6% of cases of CM occurring in childhood, according to other authors.^37^ Moreover, learning difficulties have been observed in approximately 13% of cases of pediatric mastocytosis.^13^ In contrast, neither behavioral disturbances nor neurodevelopmental disorders appear to be more common in children with mastocytosis when compared to the general population.^25^, ^29^

#### 3.3.3 Neuroimaging in mastocytosis patients with cognitive and neuropsychiatric symptoms

In this review, only Boddaert et al.^23^ analyzed both the morphology and functionality of the brain in mastocytosis patients and controls who experienced neuropsychiatric and cognitive complaints. In their study, magnetic resonance image (MRI) revealed alterations in 19 of the 39 patients, primarily consisting of White matter abnormalities (WMA)s. These abnormalities were characterized as hyperintense, smaller than 2 mm, asymmetric, and homogeneous, affecting various subcortical regions. Interestingly, they occurred with equal frequency in both cutaneous and indolent forms of mastocytosis. Furthermore, these patients exhibited increased perfusion in the putamen, as determined by MRI perfusion sequences, in comparison to patients without WMAs and healthy controls. Notably, there was a negative correlation between putamen perfusion and depression subscores.

#### 3.3.4 Other neurological manifestations in mastocytosis

Rodrigues et al^31^ conducted an examination of nine patients with different subtypes of mastocytosis who presented with neurological symptoms. These symptoms encompassed sensory manifestations (*n* = 3, limb paresthesia), motor impairments (*n* = 4, gait disorders with upper motor neuron signs), meningitis (*n* = 1) and bilateral optic neuritis (*n* = 1). Consistently, MRI scans revealed subcortical hyperintense lesions, indicating the potential involvement of mast cells in neuroinflammation within the human brain.

Furthermore, the frequency of headaches, primarily migraine, has been reported to range between 55%–69% in adult mastocytosis patients^9^, ^26^, ^28^, ^38^ and 8% in children.^37^ Notably, migraine with aura appears to be five times more frequent in mastocytosis patients compared with the general population.^28^ There is also a suggestion that some headache attacks may be triggered by MC activation flares, with a higher frequency in males and a correlation with the intensity of histaminergic symptoms.^28^ In addition, primary cough headache is present in up to 4.7% of mastocytosis patients.^28^ Finally, according to a nationwide study, the prevalence of stroke is around 4.9 per 1000 in a mastocytosis cohort,^36^ whereas MS has been reported to occur in at least 1.3% of patients.^26^

## 4 DISCUSSION

The frequency of neurological and psychocognitive manifestations, encompassing a wide array of symptoms, appears to be significantly higher in patients with mastocytosis as compared to the general population or individuals with other chronic conditions. This raises the question of whether these neurological complaints are more likely attributable to the release of proinflammatory MC mediators into the bloodstream or the infiltration of these cells into the brain. Proinflammatory cytokines and MC‐derived mediators can activate microglia through histamine receptors and induce toxicity in nervous system tissues, resulting in inflammation and subsequent neurodegeneration.^9^ Consequently, morphological brain abnormalities have been reported in nearly half of the mastocytosis patients with psychocognitive complaints. However, the absence of histological studies precludes the confirmation of potential MC infiltration into the brain.

Overall, the frequency of CI in mastocytosis has been reported to reach up to 38.6%.^24^ The aspect of cognition that is more commonly affected seems to be working memory,^9^, ^24^ a function associated with the frontal lobe,^44^ which plays a role in various higher‐level processes, including emotional regulation, social interactions, and personality.^45^ From a cohort of 39 mastocytosis patients, Boddaert et al^24^ identified a plaque‐like area in the frontal lobe resembling a low‐grade oligodendroglioma in only one patient, without any mass effect or contrast enhancement. This author also described increased perfusion in the putamen as a distinctive finding in mastocytosis patients with WMAs. It is possible that a shared pathophysiological mechanism underlies both these alterations, such as inflammation or ischemia resulting from MC activation, or disruption of the prefrontal and basal ganglia networks caused by the WMAs. Further MRI studies should be performed in mastocytosis patients with cognitive complaints to clarify these findings.

Neuropsychiatric alterations were the most common manifestations identified in this review, especially depression and anxiety. The frequency of depressive symptoms in mastocytosis ranged from 68% to 75%,^15^, ^38^ with mild depression occurring in 29%–64% of the cases,^9^, ^14^, ^15^, ^30^ and moderate to severe depression in 5%–42%.^9^, ^14^, ^15^ In contrast, the lifetime frequency of depression in the general population varies between 2% and 21%, with the highest rates found in some European countries and the lowest in some Asian countries.^46^

Concurrently, anxiety was reported in up to 27% of mastocytosis patients,^40^ stress in 42%–46%^14^, ^30^ and irritability in 54%.^9^ Anxiety is frequently related to the fear of suffering anaphylactic reactions outdoors following insects' stings, which can limit and complicate outdoor activities and social participation.^40^, ^47^ In recent years, the fear of COVID‐19 has become a new factor contributing to changes in the mental health of these patients, including increased rates of depression, anxiety and stress, potentially exacerbating MC‐related symptoms.^48^ Telomeres, whose shortening is accelerated by oxidative stress and inflammation, appeared to be shorter in individuals with mastocytosis and were positively correlated with the perceived level of stress.^14^

Factors contributing to the increased frequency of mood disorders may be closely linked to the psychological impact of disease symptoms and the sickness behavior response. Among the top 10 items reported by patients as significantly affecting their quality of life, skin manifestations, such as macules and papules, along with persistent itching, stood out.^22^ Additionally, chronic diarrhea can have adverse effects on an individual's social and sexual interests. Sickness behavior encompasses a series of natural individual responses to illness, which help the body allocate its resources toward fighting off the illness and facilitating recovery.^49^ These symptoms can include fatigue, fever, reduced activity, decreased interest in food, social withdrawal, changes in sleep patterns and increased sensitivity to pain. They are believed to be influenced by various cytokines and signaling molecules produced by the immune system.

To perform a multidisciplinary assessment of the patient, the MC Quality of Life Questionnaire^43^ is a useful tool for measuring the disease's impact on daily tasks. It has been previously reported that 42.4% of mastocytosis patients experienced a psychological burden and a mild life impairment level.^50^ However, many authors have recommended more detailed and personalized psychological interviews to complement the assessment.^51^ It is important to note that different depression diagnostic criteria and cutoffs were used in the different studies included in this review. For instance, studies employing the Hamilton‐17 scale considered scores >8 as indicative of mild depression, >17 as moderate and >24 as severe. However, some authors regarded scores >12 points as indicative of depressive symptoms,^15^ while others considered >10 points as clinically relevant.^38^ The use of more homogeneous criteria may facilitate the collection of more accurate epidemiological data regarding these neuropsychiatric manifestations in mastocytosis. Although there are no studies focused on mastocytosis to examine the benefits of psychotherapeutic interventions, there is some evidence supporting the use of cognitive behavioral therapy in conjunction with stress management interventions in other chronic disorders with similar symptom profiles.^52^

Neuropsychiatric manifestations appear to be influenced controversially by indicators of disease burden. Serum tryptase was negatively correlated with stress and depression scores,^30^ but positively correlated with impairment level^41^ and reduced quality of life.^50^ Headache severity seemed to increase with greater histaminergic symptoms^28^; however, they were also noted to be associated with antihistaminic treatments.^9^ Finally, the presence of KIT mutations negatively correlated with increased stress^14^; in contrast, inhibitory treatment with masitinib showed improvement in depression.^15^

Low serotonin levels have been reported in mastocytosis patients. Inflammatory cytokines induce the synthesis of indoleamine, shifting tryptophan metabolism toward kynurenine instead of serotonin. Additionally, this process produces end products such as quinolinic acid, which have shown neurotoxic effects and may increase cortisol levels.^30^ Patients with lower serotonin levels experienced higher rates of fatigue, migraine headaches, psychiatric symptoms, diarrhea, flushing, and abdominal and bone pain.^53^ Histamine appears to have a modulatory effect on some mnemonic systems, but its exact function in memory remains controversial.^54^ However, in some studies, all these MC‐derived mediators appeared to be independent of neuropsychiatric symptoms, leading scientists to lean toward the hypothesis that the aggressiveness of mastocytosis, rather than MC activation phenomenon, is the cause behind them.^41^ For this reason, IL‐6 has shown promise as a predictor of cognitive dysfunction, which has previously been linked to bone pain and osteoporosis.^12^ Targeting cytokines such as TNFα, IL‐1 or IL‐6 might reduce mood and CI in systemic inflammation, as has been demonstrated in other conditions like psoriasis.^55^

In children, there is limited information available on cognitive and neuropsychiatric manifestations of mastocytosis. Among 67 children diagnosed with mastocytosis, 13% showed altered cognitive test scores, leading to learning difficulties.^13^ This contrasts with the 5.4% rate of learning disabilities observed in the general pediatric population.^56^ Furthermore, irritability and headaches were highly prevalent and appeared to correlate with disease severity and skin lesions.^37^ in a cohort of 400 children with mastocytosis, the frequency of autism spectrum disorder was reported to exceed 10%,^12^ compared to the general pediatric frequency of 0.01%.^57^ This increased susceptibility to autism spectrum disorder may stem from a dysfunctional gut‐blood‐brain barrier, potentially exposing local mast cells to environmental and innate substances. This exposure can trigger the release of vasoactive, inflammatory and neurosensitizing mediators, which can impact brain function.^58^ While it is not definitively proven that attention deficit hyperactivity disorder is more prevalent in children with mastocytosis,^13^ it may be linked to these inflammatory pathways causing neuronal damage. Larger sample sizes are needed to accurately determine the true prevalence of cognitive and neuropsychiatric disorders in pediatric patients.

Several neurological disorders have been reported in mastocytosis in various studies included in this review, with headache being the most prevalent. Its frequency has been reported to be around 55%–69% in adult mastocytosis patients,^9^, ^26^, ^38^ with migraine being the predominant type.^28^ It is believed that migraine headaches are triggered by the degranulation of dural mast cells, which in turn activates the trigeminal pain pathway.^59^ This could explain why the aura phenomenon appears to be five times more common in mastocytosis than in the general population with migraines.^28^ Additionally, primary cough headache was found to be overrepresented, affecting 4.7% of mastocytosis patients in the cohort studied by Smith et al.,^28^ in contrast to the 1.2% frequency observed in the general population.^60^

The increased prevalence of MS in individuals with mastocytosis remains a subject of controversy, and confirming this association would necessitate a population‐based study. Relationships in pathophysiology have been described in the literature, linking mast cells to demyelinating lesions and noting their heightened activation in the cerebrospinal fluid of MS patients.^61^ Additionally, there have been reports of an association between the overall MC burden and susceptibility to experimental immune encephalitis.^62^ Further insights into the relationship between mastocytosis and MS can potentially be gained through neuroimaging and histological analysis of patients with both conditions.

Interestingly, a similar prevalence of neurological and psychiatric symptoms has been reported in the two main subtypes of mastocytosis, cutaneous and systemic, in both adults^9^ and children.^29^ Given that diagnostic misclassification does not appear to be the primary reason for this finding, it may suggest that inflammatory mediators, rather than MC infiltration of the nervous system, are the primary pathophysiological mechanisms involved. However, the only study that included MRI findings found abnormalities at a comparable rate in both groups, with 5 out of 12 cases in the cutaneous subtype (42%) and 14 out of 27 cases in the systemic subtype (52%).^23^ This raises the question of whether apparently isolated cutaneous forms may be associated with involvement of other organs or if brain lesions may result from systemic inflammatory mediators. However, it is important to note that the limited population size avoids drawing solid conclusions. More neuroimaging studies with larger cohorts of both cutaneous and SM forms are needed to clarify this knowledge gap.

Similarly, a nationwide study calculated that the risk of stroke in mastocytosis patients is 1.6 times higher compared to controls.^36^ This increased risk may be attributed to the heightened cardiovascular susceptibility in these patients, primarily stemming from their proinflammatory state. Furthermore, endothelial dysfunction has been previously observed in mastocytosis, characterized by reduced flow‐mediated dilatation and elevated serum vascular endothelial growth factor levels, even in the absence of atherosclerosis or systemic inflammation. These findings have been linked to disease severity.^63^

Other neurological conditions have been scarcely described in mastocytosis patients in various case reports. These conditions include parkinsonism,^64^ chorea,^65^ seizures,^66^ syncope,^67^ hypotensive shock,^68^ encephalopathy due to cerebral hypoxia,^69^ peripheral neuropathy,^70^, ^71^, ^72^, ^73^ neurosensory deafness,^74^ meningeal irritation and compressive myelopathy due to BM infiltration,^71^ conus medullaris syndrome,^75^ intraspinal tumo^76^ and hematomas,^77^ and myotonic dystrophy.^78^ All these reports were excluded from this review because of the lack of larger cohorts providing data applicable to the general population.

### 4.1 Limitations

The main limitations of this systematic review arise from the heterogeneity of the study protocols and the variations in the neurocognitive and psychiatric tests used. Additionally, the inclusion of different subtypes of mastocytosis across studies makes it challenging to draw precise conclusions regarding which form of the disease exhibits more neurological alterations. Furthermore, when assessing the prevalence of specific conditions, the studies not only employ diverse assessment scales but also apply different diagnostic cutoffs and criteria for disease severity. Finally, the available neurological system imaging and histological data are insufficient to provide comprehensive insights into the underlying pathophysiological mechanisms of these abnormalities.

## 5 CONCLUSION

This systematic review highlights a significant percentage of mastocytosis patients experiencing cognitive, neurological and neuropsychiatric alterations. This underscores the importance of early detection and effective management of these symptoms to enhance the quality of life of these patients. Further studies should be designed to compare the cognitive functions and neuropsychiatric symptoms of mastocytosis patients with controls, using more comprehensive evaluations and standardized tests to better understand their relationship. Particularly, the field of neuroimaging remains underexplored in this disease. Expanding imaging databases and conducting a histological analysis of lesions could provide valuable insights into our understanding of mastocytosis pathophysiology.

## AUTHOR CONTRIBUTIONS

**Elena Sagües‐Sesé**: Data curation (equal); methodology (equal); writing—original draft (equal). **Natalia García‐Casares**: Conceptualization (equal); methodology (equal); supervision (equal); writing—review and editing (equal). **Ivan Álvarez‐Twose**: Conceptualization (equal); methodology (equal); supervision (equal); writing—review and editing (equal).

## CONFLICT OF INTEREST STATEMENT

The authors have no conflicts of interest to declare.

## Data Availability

The data that support the findings of this study are available on request from the authors.
